# Supplementary material for: Tropomyosin 1-I/C coordinates kinesin-1 and dynein motors during oskar mRNA transport
Source: Nat Struct Mol Biol. 2024 Jan 31;31(3):476–88. doi: 10.1038/s41594-024-01212-x (PMC10948360; doi:10.1038/s41594-024-01212-x)
Supplement: Supplementary file 1 — Supplementary Figs. 1–5, Tables 1–6 and RNA sequences used in this study. [file 41594_2024_1212_MOESM1_ESM.pdf]

nature structural & molecular biology

Article https://doi.org/10.1038/s41594-024-01212-x

Tropomyosin 1-I/C coordinates kinesin-1 and dynein motors during *oskar* mRNA transport

In the format provided by the authors and unedited

Supplementary Information

Tropomyosin 1-I/C co-ordinates kinesin-1 and dynein motors during *oskar* mRNA transport

Simone Heber[1†], Mark A. McClintock[2†], Bernd Simon[3,4], Eve Mehtab[1], Karine Lapouge[5], Janosch Hennig[3,6], Simon L. Bullock[2*], Anne Ephrussi[1*]

1 Developmental Biology Unit, European Molecular Biology Laboratory, 69117 Heidelberg, Germany

2 Division of Cell Biology, MRC Laboratory of Molecular Biology, Cambridge CB2 0QH, United Kingdom

3 Structural and Computational Biology Unit, European Molecular Biology Laboratory, 69117 Heidelberg, Germany

4 Department of Molecular Biology and Biophysics, University of Connecticut Health Center, Farmington, CT, USA

5 Protein Expression and Purification Core Facility, European Molecular Biology Laboratory, 69117 Heidelberg, Germany

6 Biochemistry IV, Biophysical Chemistry, University of Bayreuth, 95447 Bayreuth, Germany

†These authors contributed equally to this work

*Correspondence: sbullock@mrc-lmb.cam.ac.uk, anne.ephrussi@embl.org

Supplementary Figures

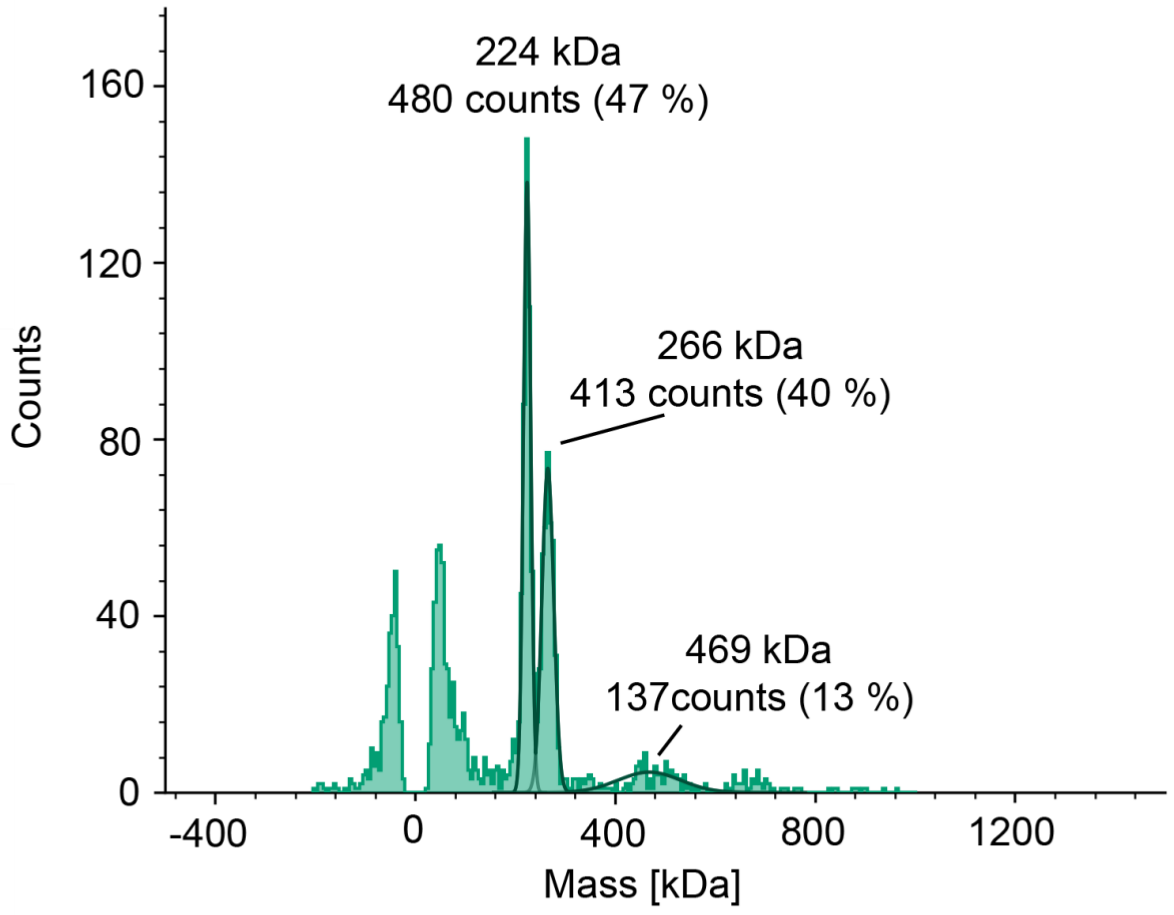

Figure S1: Khc and Tm1 form a complex *in vitro*. Mass photometry measurements of the Khc FL-Tm1 FL complex at 20 nM concentration. The sample comprises a mixture of 47% particles of 224 kDa, corresponding to the Khc dimer (theoretical MW = 221.2 kDa), 40% particles of 266 kDa, corresponding to a Khc dimer bound to a Tm1 monomer (theoretical MW = 269.2 kDa), and 13% of the particles represent larger species. This indicates that in solution, ~50% of Khc is bound to Tm1 at steady-state at concentrations below the K_D, confirming the 2:1 stoichiometry of the Khc-Tm1 complex[1].

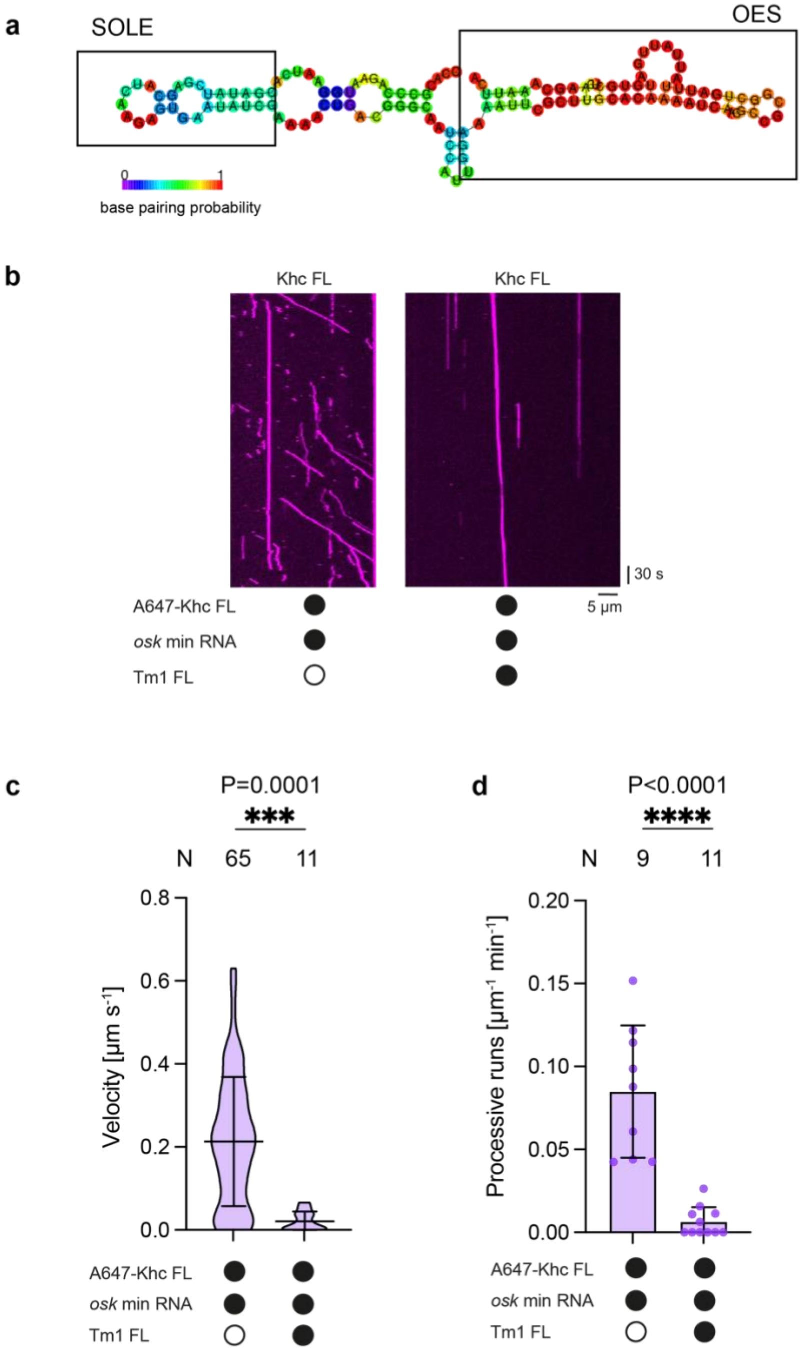

Figure S2: The SOLE does not render Khc resistant to inhibition by Tm1. a RNAfold[2] MFE prediction of *osk* min RNA containing both the OES and SOLE stem loops. The structures of these localization elements are predicted to be preserved in the *osk* min construct. **b** Kymographs showing the motile behavior of Khc FL in the presence or absence of Tm1 FL. Microtubule plus and minus ends are oriented toward the right and left of each kymograph, respectively. **c, d** Velocity of motile Khc FL (c) and frequency of processive Khc FL movements (d) in the presence or absence of Tm1 FL. Quantification is from one representative experiment of five in which strong inhibition of Khc motility by Tm1 in the presence of *osk* min was observed. For all plots, the mean ± SD is shown and is derived from 11-65 individual complexes (c; N) or 9-11 microtubules (d; N) from 2-3 imaging chambers per condition. In all panels, black or white circles indicate the presence or absence of indicated components, respectively. Unlabeled *osk* min was included in all experiments. Statistical significance was determined by unpaired two-tailed t-tests with Welch's correction using N values (total number of individual complexes (c) or total number of microtubules (d)).

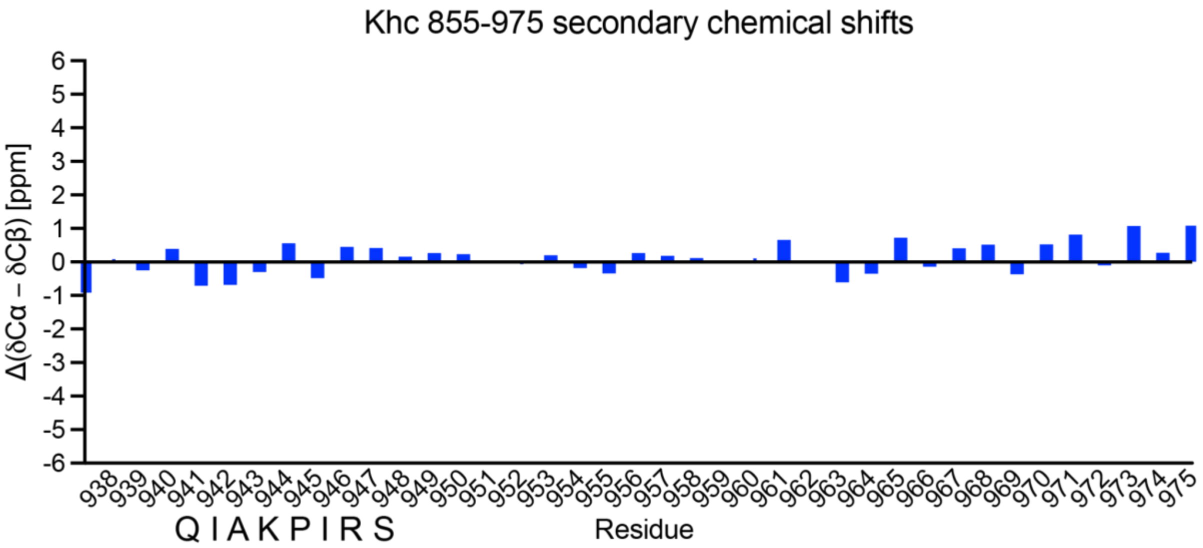

Figure S3: Secondary chemical shifts of Khc 855-975. Secondary structure is not detected in the part of the construct visible by NMR (even transient secondary structure elements should give values > 1).

a

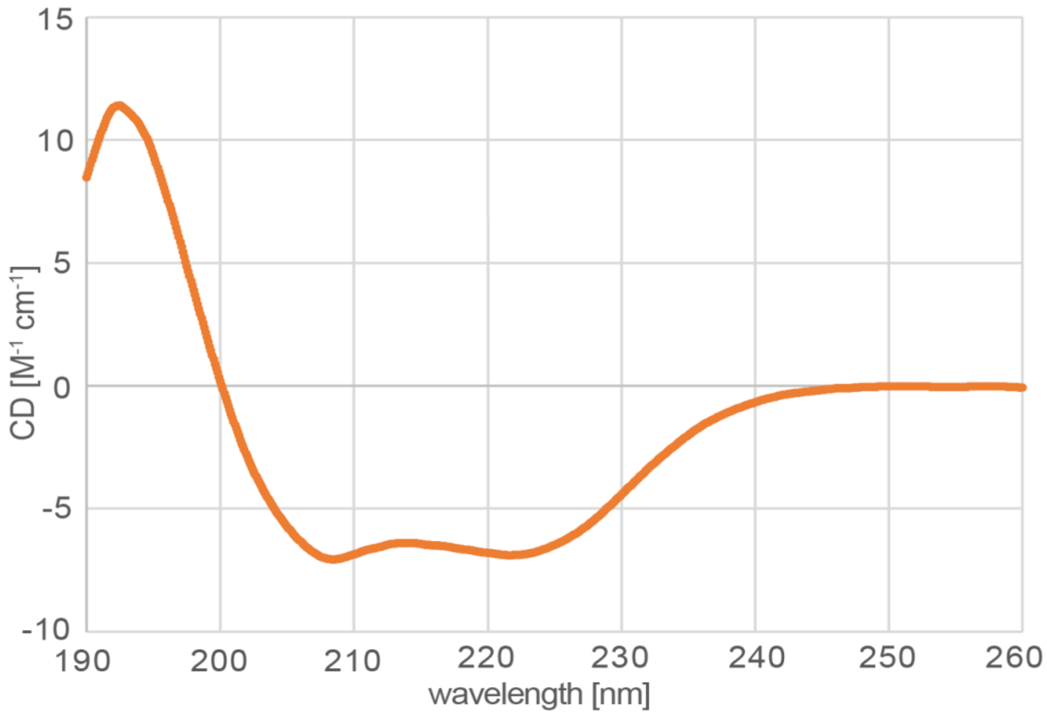

b

% Helix	% Strand	% Disordered
67.8	7.5	27.6

Figure S4: CD spectrum of Khc 855-975. a The CD spectrum of Khc 855-975 shows high α-helical content with minima at 208 nm and 222 nm that have a ratio of 0.97, consistent with the presence of a coiled-coil structure within the protein[3]. **b** Secondary structure content of Khc 855-975 calculated according to the CDSSTR, Selcon3 and Contin-LL methods using the Dichroweb server.

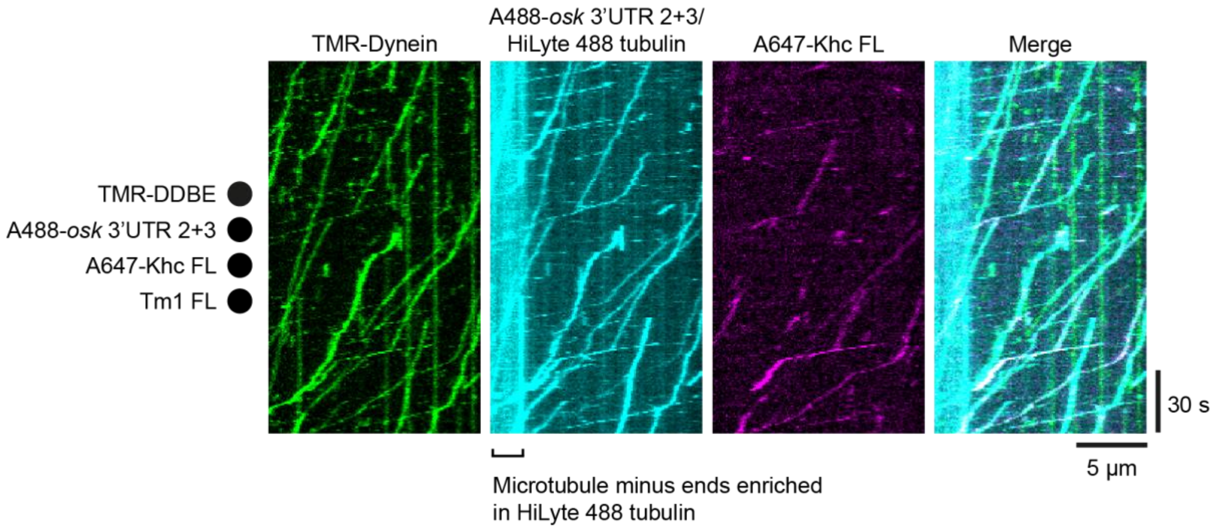

Figure S5: Khc moves toward microtubule minus ends with DDBE and *osk* RNA. Kymographs showing the Tm1 FL-induced co-translocation of dynein, *osk* 3′UTR 2+3 RNA, and Khc FL toward microtubule minus ends, which are labeled by enrichment of fluorescent (HiLyte 488) tubulin dimers. Microtubule plus and minus ends are oriented toward the right and left of each kymograph, respectively. Black circles indicate the presence of indicated components.

Supplementary Tables

	MT binding events [min^{-1} μm^{-1}]	Processive events [min^{-1} μm^{-1}]	Fraction of processive events	Mean velocity [$\mu m\ s^{-1}$]
**Khc FL**	0.30 ± 0.26 (N=59 MTs, 5 independent experiments)	0.24 ± 0.18 (N=59 MTs, 5 independent experiments)	0.77 ± 0.12 (N=59 MTs, 5 independent experiments)	0.37 ± 0.28 (N=748 complexes, 5 independent experiments)
**Khc FL** + **Tm1 FL**	0.19 ± 0.16 (N=38 MTs, 5 independent experiments)	0.10 ± 0.16 (N=38 MTs, 5 independent experiments)	0.45 ± 0.29 (N=38 MTs, 5 independent experiments)	0.13 ± 0.15 (N=205 complexes, 5 independent experiments)
**Khc910**	0.59 ± 0.23 (N=16 MTs, 2 independent experiments)	0.59 ± 0.23 (N=16 MTs, 2 independent experiments)	0.97 ± 0.01 (N=16 MTs, 2 independent experiments)	0.55 ± 0.22 (N=755 complexes, 2 independent experiments)
+ **Tm1 FL**	0.68 ± 0.19 (N=21 MTs, 2 independent experiments)	0.68 ± 0.19 (N=21 MTs, 2 independent experiments)	0.98 ± 0.02 (N=21 MTs, 2 independent experiments)	0.53 ± 0.24 (N=2185 complexes, 2 independent experiments)
**Khc940**	1.21 ± 0.57 (N=28 MTs, 3 independent experiments)	1.19 ± 0.55 (N=28 MTs, 3 independent experiments)	0.98 ± 0.02 (N=28 MTs, 3 independent experiments)	0.47 ± 0.20 (N=1802 complexes, 3 independent experiments)
+ **Tm1 FL**	0.63 ± 0.34 (N=26 MTs, 3 independent experiments)	0.39 ± 0.32 (N=26 MTs, 3 independent experiments)	0.61 ± 0.22 (N=26 MTs, 3 independent experiments)	0.12 ± 0.10 (N=662 complexes, 3 independent experiments)
**KhcΔAMB**	0.12 ± 0.11 (N=36 MTs, 3 independent experiments)	0.03 ± 0.02 (N=36 MTs, 3 independent experiments)	0.61 ± 0.30 (N=36 MTs, 3 independent experiments)	0.52 ± 0.36 (N=162 complexes, 3 independent experiments)
+ **Tm1 FL**	0.13 ± 0.07 (N=53 MTs, 3 independent experiments)	0.09 ± 0.05 (N=53 MTs, 3 independent experiments)	0.82 ± 0.17 (N=53 MTs, 3 independent experiments)	0.58 ± 0.25 (N=811 complexes, 3 independent experiments)

Table S1: Motility parameters of Khc FL, Khc910, Khc940 and KhcΔAMB in the presence of *osk* 3'UTR ± Tm1 FL (Related to Figures 1, 2, Extended Data Figures 1-3). Mean ± SD are shown.

	MT binding events [min^{-1} μm^{-1}]	Processive events [min^{-1} μm^{-1}]	Fraction of processive events	Mean velocity [μm s^{-1}]
**Khc FL**	0.23 ± 0.08 (N=25 microtubules, 2 independent experiments)	0.16 ± 0.06 (N=25 microtubules, 2 independent experiments)	0.72 ± 0.28 (N=25 microtubules, 2 independent experiments)	0.33 ± 0.24 (N=398 complexes, 2 independent experiments)
+ **Tm1 1-335**	0.13 ± 0.03 (N=34 microtubules, 2 independent experiments)	0.06 ± 0.04 (N=34 microtubules, 2 independent experiments)	0.35 ± 0.19 (N=34 microtubules, 2 independent experiments)	0.15 ± 0.17 (N=341 complexes, 2 independent experiments)

Table S2: Motility parameters of Khc FL in the presence of *osk* 3'UTR ± Tm1 1-335 (Related to Figure 3). Mean ± SD are shown.

	Total dynein binding frequency [μm^{-1} min^{-1}]	Processive dynein frequency [μm^{-1} min^{-1}]	Processive dynein fraction	Mean dynein velocity [μm s^{-1}]	Dynein run length [µm]
**DDBE-*osk* 3′UTR 2+3 -Tm1 FL**	1.16 ± 0.20 N=20 microtubules (2152 complexes, 2 independent experiments)	0.67 ± 0.14 N=20 microtubules (1277 complexes, 2 independent experiments)	0.58 ± 0.07 N=20 microtubules (2152 complexes, 2 independent experiments)	0.138 (median) 0.071-0.467 (IQ range) N=1277 complexes (2 independent experiments)	2.87 (median) 1.58-5.64 (IQ range) N=1277 complexes (2 independent experiments)
**DDBE-*osk* 3′UTR 2+3 +Tm1 FL**	1.27 ± 0.24 N=20 microtubules (2207 complexes, 2 independent experiments)	0.75 ± 0.19 N=20 microtubules (1295 complexes, 2 independent experiments)	0.58 ± 0.06 N=20 microtubules (2207 complexes, 2 independent experiments)	0.162 (median) 0.069-0.502 (IQ range) N=1295 complexes (2 independent experiments)	3.01 (median) 1.61-5.71 (IQ range) N=1295 complexes (2 independent experiments)
**DDBE-*osk* 3′UTR 2+3 +Tm1 FL (Tm1-bound only)**	N/A	N/A	N/A	0.166 (median) 0.083-0.422 (IQ range) N=211 complexes (2 independent experiments)	N/A

Table S3: Motility parameters of dynein (representing DDBE-*osk* 3′UTR 2+3) in presence or absence of Tm1 FL (Related to Extended Data Figure 7). Mean ± SD are shown unless otherwise indicated.

	Frequency of Khc transport events toward plus ends [μm^{-1} min^{-1}]	Frequency of Khc transport events toward minus ends [μm^{-1} min^{-1}]	Fraction of Khc transport events toward plus ends	Fraction of Khc transport events toward minus ends	Mean Khc-bound dynein velocity [μm s^{-1}]	Distance traveled by Khc toward minus ends [µm]
**DDBE-*osk* 3′UTR 2+3 +Khc FL -Tm1 FL**	0.005 ± 0.008 N=66 microtubules (40 complexes, 3 independent experiments)	0.019 ± 0.018 N=66 microtubules (138 complexes, 3 independent experiments)	0.198 ± 0.230 N=53 microtubules (178 complexes, 3 independent experiments)	0.802 ± 230 N=53 microtubules (178 complexes, 3 independent experiments)	0.126 (median) 0.066-0.242 (IQ range) N=158 complexes (3 independent experiments)	1.97 (median) 1.21-3.79 (IQ range) N=158 complexes (3 independent experiments)
**DDBE-*osk* 3′UTR 2+3 +Khc FL +Tm1 FL**	0.007 ± 0.013 N=60 microtubules (35 complexes, 3 independent experiments)	0.194 ± 0.072 N=60 microtubules (1167 complexes, 3 independent experiments)	0.031 ± 0.055 N=60 microtubules (1202 complexes, 3 independent experiments)	0.969 ± 0.055 N=60 microtubules (1202 complexes, 3 independent experiments)	0.220 (median) 0.097-0.463 (IQ range) N=1259 complexes (3 independent experiments)	3.15 (median) 1.86-5.99 (IQ range) N=1259 complexes (3 independent experiments)
**DDBE-*osk* 3′UTR 2+3 +Khc940 -Tm1 FL**	0.144 ± 0.073 N=86 microtubules (1219 complexes, 3 independent experiments)	0.050 ± 0.034 N=86 microtubules (469 complexes, 3 independent experiments)	0.731 ± 0.163 N=86 microtubules (1688 complexes, 3 independent experiments)	0.269 ± 0.163 N=86 microtubules (1688 complexes, 3 independent experiments)	0.070 (median) 0.036-0.166 (IQ range) N=469 complexes (3 independent experiments)	2.36 (median) 1.37-4.62 (IQ range) N=469 complexes (3 independent experiments)
**DDBE-*osk* 3′UTR 2+3 +Khc940 +Tm1 FL**	0.100 ± 0.065 N=88 microtubules (719 complexes, 3 independent experiments)	0.138 ± 0.073 N=88 microtubules (1076 complexes, 3 independent experiments)	0.417 ± 0.174 N=88 microtubules (1795 complexes, 3 independent experiments)	0.583 ± 0.174 N=88 microtubules (1795 complexes, 3 independent experiments)	0.078 (median) 0.037-0.169 (IQ range) N=1076 complexes (3 independent experiments)	2.47 (median) 1.58-4.41 (IQ range) N=1076 complexes (3 independent experiments)

Table S4: Motility parameters of dynein (representing DDBE-*osk* 3′UTR 2+3) and Khc FL or Khc940 in presence or absence of Tm1 FL (Related to Figure 6 and Extended Data Figure 8). Mean ± SD are shown unless otherwise indicated.

SAXS sample details

Sample	Khc	Khc-Tm1
Organism	*Drosophila melanogaster*	*Drosophila melanogaster*
Source	Sf21 insect cells	Sf21 insect cells
Description	Kinesin heavy chain dimer (Uniprot: P17210 (1-975))	Complex of Kinesin heavy chain (Uniprot: P17210 (1-975)) and Tropomyosin1 isoform I/C (Uniprot: Q95TA3 (1-441))
Molecular mass *M* from chemical composition (kDa)	221	269 (2:1 complex stoichiometry)
Concentration (range/values) measured and method	0.5 mg mL^{-1}	0.5 mg mL^{-1}
Solvent composition	25 mM HEPES/KOH pH 7.3, 150 mM KCl, 1 mM $MgCl_2$, 2 mM DTT	25 mM HEPES/KOH pH 7.3, 150 mM KCl, 1 mM $MgCl_2$, 2 mM DTT

SAXS data collection parameters

Source, instrument and description or reference	BM29 ESRF Grenoble with Pilatus2M
Wavelength (Å)	0.9919

sample-to-detector distance (m)	2.813
q-measurement range (nm^{-1})	0.043 – 5.179
Method for monitoring radiation damage	frame-by-frame comparison
Exposure time (s), number of exposures	1.0 x 10
Sample configuration	sample changer with flow through capillary
Sample temperature (ºC)	20

Software employed for SAXS data reduction, analysis and interpretation

SAXS data reduction to sample–solvent scattering	ATSAS 2.8.4-1
Atomic structure modeling	CNS-1.2

Atomistic modeling

Method	Crysol 2.8.3	
q-range for fitting	0.043-5.00	
χ^2 value	3.071	1.684
predicted R_g	94.88	112.3

Table S5: Reporting table for SAXS data acquisition, showing sample details, data analysis, model fitting and software used.

Plasmids

CONSTRUCTS	RELEVANT INFORMATION	SOURCE
pFastBacDual-HisSUMO-SNAP-3C-Khc	Amp^r, p10/Polyhedrin promoter	This study
pFastBacDual-HisSUMO-SNAP-3C-Khc910	Amp^r, p10/Polyhedrin promoter	This study
pFastBacDual-HisSUMO-SNAP-3C-Khc940	Amp^r, p10/Polyhedrin promoter	This study
pFastBacDual-HisSUMO-SNAP-3C-Khc ΔAMB	Amp^r, p10/Polyhedrin promoter	This study
pFastBacDual-HisSUMO-SNAP-3C-Khc-Tm1	Amp^r, p10/Polyhedrin promoter	This study
pGEX6.1-Khc 855-975	Amp^r, tac promoter	This study
pGEX6.1-Khc 1-365	Amp^r, tac promoter	This study
pETM11-His6-SUMO-SNAP-Tm1-FL	Kan^r, T7 promoter, lac operator	This study
pETM11-His6-SNAP-SUMO-Tm1 1-335	Kan^r, T7 promoter, lac operator	This study

pETM11-His6-SUMO-Tm1-FL	Kan^r, T7 promoter, lac operator	Ref. 1
pETM11-His6-SUMO-Tm1 1-335	Kan^r, T7 promoter, lac operator	Ref. 1
pDyn1 (pAceBac1-ZZ-TEV-SNAPf-Dynein heavy chain - DYNC1H1)	Gen^r, Polyhedrin promoter	Ref. 4
pDyn2 (pIDC-Dynein intermediate chain - DYNC1/2, Dynein light intermediate chain - DYNC1LI2, Tctex - DYNLT1, Robl - DYNLRB1, LC8 - DYNLL1)	Chlor^r, Polyhedrin promoter	Ref. 4
pAceBac1-Egl-TEV-ZZ	Gen^r, Polyhedrin promoter	Ref. 5
pIDC-BicD	Chlor^r, Polyhedrin promoter	Ref. 5

Table S6: Plasmids used in this study.

RNA sequences

osk 3′UTR

GUUGGGUUCUUAAUCAAGAUACAUAUAUGCAAAUUUUGACUGGGCUGGCACC
GGAACCGACAAAAUAAGAACUUUUUGAUGAUUUUACGAUUUACGCUGAUGGA
UCGCUGCUUUUACGGAAUUCGCUUAGUUUUAAUAUGUUUUAUAUGUAGUAUG
UUCUCUGUCUUUGUUUAUUUAUAUGUUCGUGCACUUGUCCUAGUCCAUUAUU
GUAUAUUAUUGUGUGUUUUGUGUUCUAUGUUAGAUUUAAACUUCUCAAUUUU
UCGCUGUCUGUGAUUUGUUUUGCCAAUGCCAUUGAUUUUCUGCACACUUUU
UGCUGCUAUCCCAAAAGCUGUGUAAAAUAAUCAAAUGCAAAAUAAGCGCAAGC
AGCUGAAAACUUCUCUUCAAACUUUUCCGCUUUUCCCAAAACCAUUUUGCUU
UGAAAUCUGUUUUUACCAAAUCAGAUUAAACUGCAAAAUGGAACUUAAAUGCA
AAUCAUUGCAAUGCUUAUAAACUGUUUUUUGUUCUAUAUACUUUUGUGUGGG

UCAAAAUUCGGCAUGCUCCUGUAUCACACAACCUGCCACUUGCCCUUAAAAA
GAAGGGCGCAGUGGGCGUGGUACGUAUACAUAUGAGCCAUGCUGCAUUUUG
GCCGUAAUGAAAAUGCACUGCUUUACUUGGAAAAUUCGCUUGCACAAAAUCA
ACGCCGCGGCUGAUUUAUUAUUGAUGUGCUCAAGCAAAUUCAAGUGAAGCAU
UUGCGCGAUUUUCGUCUUUCUGUUUCCGUUUGCAAAAAAGUUUAUAAAAUGC
UUACACUCUGCUGCAGACACGCCAACCGGAAGUGCGCACUAAGCGCUUGUUU
GUAGCACAGUGUAGAAUUCUGGCGUAAUUUACAGCUCUACUUUAAAGUCUUC
UAGAUAGCUAUCUACUAUUUAUAAACUUAUUUAUUGUCUUGAAUGUAUGUUA
AUUGUAUGUAUUGAUGGUGAUCACGUUUUUUUGUCCUAUAACAAGCUGCAA
UGUAAAAUCCAAAAAAAAAAUGAAAAAAUAAAAUAAAAGGGAAAUCAAUGAUUU
UGUUUGCUCUAAGCUAUGUUCAAAGUAACUGG

Total length: 1074 nt

***osk* min**

CCACGCCCAGAAUGGAAUCACGAUAUCGAGCAUCAAGAGUGAAUAUCGAAAA
CUGACGGGCAAUCCAUUGGAAAAUUCGCUUGCACAAAAUCAACGCCGCGGCU
GAUUUAUUAUUGAUGUGCUCAAGCAAAUUCA

Total length: 135 nt

SOLE

OES

***osk* 3′UTR regions 2+3**

UGUUCUAUAUACUUUUGUGUGGGUCAAAAUUCGGCAUGCUCCUGUAUCACAC
AACCUGCCACUUGCCCUUAAAAAGAAGGGCGCAGUGGGCGUGGUACGUAUAC
AUAUGAGCCAUGCUGCAUUUUGGCCGUAAUGAAAAUGCACUGCUUUACUUGG
AAAAUUCGCUUGCACAAAAUCAACGCCGCGGCUGAUUUAUUAUUGAUGUGCU
CAAGCAAAUUCAAGUGAAGCAUUUGCGCGAUUUUCGUCUUUCUGUUUCCGUU
UGCAAAAAAGUUUAUAAAAUGCUUACACUCUGCUGCAGACACGCCAACCGGA
AGUGCGCACUAAGCGCUUGUUUGUAGCACAGUGUAGAAUUCUGGCGUAAUU
UACAGCUCUACUUUAAAGUCUUCUAGAUAGCUAUCUACUAUUUAUAAACUUAU
UUAUUGUCUUGAAUGUAUGUUAAUUGUAUGUAUUGAUGGUGAUCACGUUUUU
UUUGUCCUAUAACAAGCUGCAAUGUAAAAUCCAAAAAAAAAAUGAAAAAAUAAA
AUAAAAGG

Total length: 530 nt

Supplement references

1. Dimitrova-Paternoga L, Jagtap PKA, Cyrklaff A, Vaishali, Lapouge K, Sehr P, et al. Molecular basis of mRNA transport by a kinesin-1-atypical tropomyosin complex. Genes Dev. 2021 Jul 1;35(13–14):976–91.
2. Gruber AR, Lorenz R, Bernhart SH, Neuböck R, Hofacker IL. The Vienna RNA websuite. Nucleic Acids Res. 2008 Jul 1;36(Web Server issue):W70-4.
3. Lau SY, Taneja AK, Hodges RS. Synthesis of a model protein of defined secondary and quaternary structure. Effect of chain length on the stabilization and formation of two-stranded alpha-helical coiled-coils. J Biol Chem. 1984 Nov 10;259(21):13253–61.
4. Schlager MA, Hoang HT, Urnavicius L, Bullock SL, Carter AP. In vitro reconstitution of a highly processive recombinant human dynein complex. EMBO J. 2014 Sep 1;33(17):1855–68.
5. McClintock MA, Dix CI, Johnson CM, McLaughlin SH, Maizels RJ, Hoang HT, et al. RNA-directed activation of cytoplasmic dynein-1 in reconstituted transport RNPs. eLife 7:e36312 (2018).
