# Supplementary material for: Tropomyosin 1-I/C coordinates kinesin-1 and dynein motors during oskar mRNA transport
Source: Nat Struct Mol Biol. 2024 Jan 31;31(3):476–88. doi: 10.1038/s41594-024-01212-x (PMC10948360; doi:10.1038/s41594-024-01212-x)
Supplement: Supplementary file 2 — Reporting Summary [file 41594_2024_1212_MOESM2_ESM.pdf]

Corresponding author(s): Anne Ephrussi and Simon Bullock

Last updated by author(s): Sep 29, 2023

# Reporting Summary

Nature Portfolio wishes to improve the reproducibility of the work that we publish. This form provides structure for consistency and transparency in reporting. For further information on Nature Portfolio policies, see our Editorial Policies and the Editorial Policy Checklist.

## Statistics

For all statistical analyses, confirm that the following items are present in the figure legend, table legend, main text, or Methods section.

| n/a | Confirmed | |
|---|---|---|
| ☐ | ☒ | The exact sample size (*n*) for each experimental group/condition, given as a discrete number and unit of measurement |
| ☐ | ☒ | A statement on whether measurements were taken from distinct samples or whether the same sample was measured repeatedly |
| ☐ | ☒ | The statistical test(s) used AND whether they are one- or two-sided<br>*Only common tests should be described solely by name; describe more complex techniques in the Methods section.* |
| ☐ | ☒ | A description of all covariates tested |
| ☐ | ☒ | A description of any assumptions or corrections, such as tests of normality and adjustment for multiple comparisons |
| ☐ | ☒ | A full description of the statistical parameters including central tendency (e.g. means) or other basic estimates (e.g. regression coefficient) AND variation (e.g. standard deviation) or associated estimates of uncertainty (e.g. confidence intervals) |
| ☐ | ☒ | For null hypothesis testing, the test statistic (e.g. *F*, *t*, *r*) with confidence intervals, effect sizes, degrees of freedom and *P* value noted<br>*Give P values as exact values whenever suitable.* |
| ☒ | ☐ | For Bayesian analysis, information on the choice of priors and Markov chain Monte Carlo settings |
| ☒ | ☐ | For hierarchical and complex designs, identification of the appropriate level for tests and full reporting of outcomes |
| ☒ | ☐ | Estimates of effect sizes (e.g. Cohen's *d*, Pearson's *r*), indicating how they were calculated |

*Our web collection on statistics for biologists contains articles on many of the points above.*

## Software and code

Policy information about availability of computer code

| | |
|---|---|
| Data collection | Leica LAS X, Micromanager<br>AcquireMP (Refeyn Ltd; version 2.5.0)<br>Topspin 3.5 (Bruker)<br>ESRF BM29 automatic processing pipeline |
| Data analysis | FIJI (ImageJ2 version 2.9.0/1.53t)<br>GraphPad Prism version 9.1.1<br>DiscoverMP (Refeyn Ltd; version 2.5.0)<br>NMRPipe v10.9, Cara 1.9.1, NMRView 5.0.4<br>ATSAS 3.0.4, CNS-1.2<br>CRYSOL 2.8.3 |

For manuscripts utilizing custom algorithms or software that are central to the research but not yet described in published literature, software must be made available to editors and reviewers. We strongly encourage code deposition in a community repository (e.g. GitHub). See the Nature Portfolio guidelines for submitting code & software for further information.

## Data

Policy information about availability of data

All manuscripts must include a data availability statement. This statement should provide the following information, where applicable:

- Accession codes, unique identifiers, or web links for publicly available datasets
- A description of any restrictions on data availability
- For clinical datasets or third party data, please ensure that the statement adheres to our policy

Source data are provided with this paper. The backbone chemical shift assignment of Khc 855-975 has been deposited to the BMRB under the accession code 51888 and the SAXS data have been submitted to SASBDB (IDs SASDRD8 and SASDRE8).

## Human research participants

Policy information about studies involving human research participants and Sex and Gender in Research.

| | |
|---|---|
| Reporting on sex and gender | n/a |
| Population characteristics | n/a |
| Recruitment | n/a |
| Ethics oversight | n/a |

Note that full information on the approval of the study protocol must also be provided in the manuscript.

# Field-specific reporting

Please select the one below that is the best fit for your research. If you are not sure, read the appropriate sections before making your selection.

☒ Life sciences ☐ Behavioural & social sciences ☐ Ecological, evolutionary & environmental sciences

For a reference copy of the document with all sections, see nature.com/documents/nr-reporting-summary-flat.pdf

# Life sciences study design

All studies must disclose on these points even when the disclosure is negative.

| | |
|---|---|
| Sample size | Typical experiments yielded dozens to hundreds of single-molecule observations from dozens of individual microtubules. These experiments were independently replicated at least twice and with a greater number of replicates in the vast majority of replicates. |
| Data exclusions | Poorly resolved microtubules or microtubule bundles were not analyzed as they obscure and distort motor metrics. Apparent interactions of labeled protein/RNA with microtubules were excluded from analysis if they failed to meet the defining criteria for any given metric as defined in Methods. |
| Replication | All results were replicated both within the paper using identical experimental conditions and independently under varying conditions not described in the study. |
| Randomization | Overall, randomization was not applicable as biophysical metrics were gathered for individual molecules/purified components. To prevent weighting of these metrics in favor of replicates that stochastially had more microtubules in a field of view than others, microtubules in more densely coated cover slips were randomly selected for analysis prior to viewing the behavior of motor components. |
| Blinding | NMR and SAXS data of purified proteins cannot be blinded as they are measuring innate properties of these components and not different between two experimental conditions. For single-molecule experiments, fields view and microtubules for imaging were selected before viewing channels for other labeled protein/RNA components. |

# Reporting for specific materials, systems and methods

We require information from authors about some types of materials, experimental systems and methods used in many studies. Here, indicate whether each material, system or method listed is relevant to your study. If you are not sure if a list item applies to your research, read the appropriate section before selecting a response.

## Materials & experimental systems

| n/a | Involved in the study |
| --- | --- |
| ☒ | ☐ Antibodies |
| ☐ | ☒ Eukaryotic cell lines |
| ☒ | ☐ Palaeontology and archaeology |
| ☒ | ☐ Animals and other organisms |
| ☒ | ☐ Clinical data |
| ☒ | ☐ Dual use research of concern |

## Methods

| n/a | Involved in the study |
| --- | --- |
| ☒ | ☐ ChIP-seq |
| ☒ | ☐ Flow cytometry |
| ☒ | ☐ MRI-based neuroimaging |

# Eukaryotic cell lines

Policy information about cell lines and Sex and Gender in Research

| | |
| --- | --- |
| Cell line source(s) | Sf21 (ECACC 05022801): Derived from pupal ovarian tissue of Spodoptera frugiperda<br>Sf9 (ECACC 89070101): Derived from pupal ovarian tissue of Spodoptera frugiperda |
| Authentication | The cell lines were not authenticated. |
| Mycoplasma contamination | Insect cell lines used for production of recombinant proteins were not tested for Mycoplasma contamination. |
| Commonly misidentified lines<br>(See ICLAC register) | No commonly misidentified cell lines were used in the study. |
