# Supplementary figures and images for: Tropomyosin 1-I/C coordinates kinesin-1 and dynein motors during oskar mRNA transport
Source: Nat Struct Mol Biol. 2024 Jan 31;31(3):476–88. doi: 10.1038/s41594-024-01212-x (PMC10948360; doi:10.1038/s41594-024-01212-x)

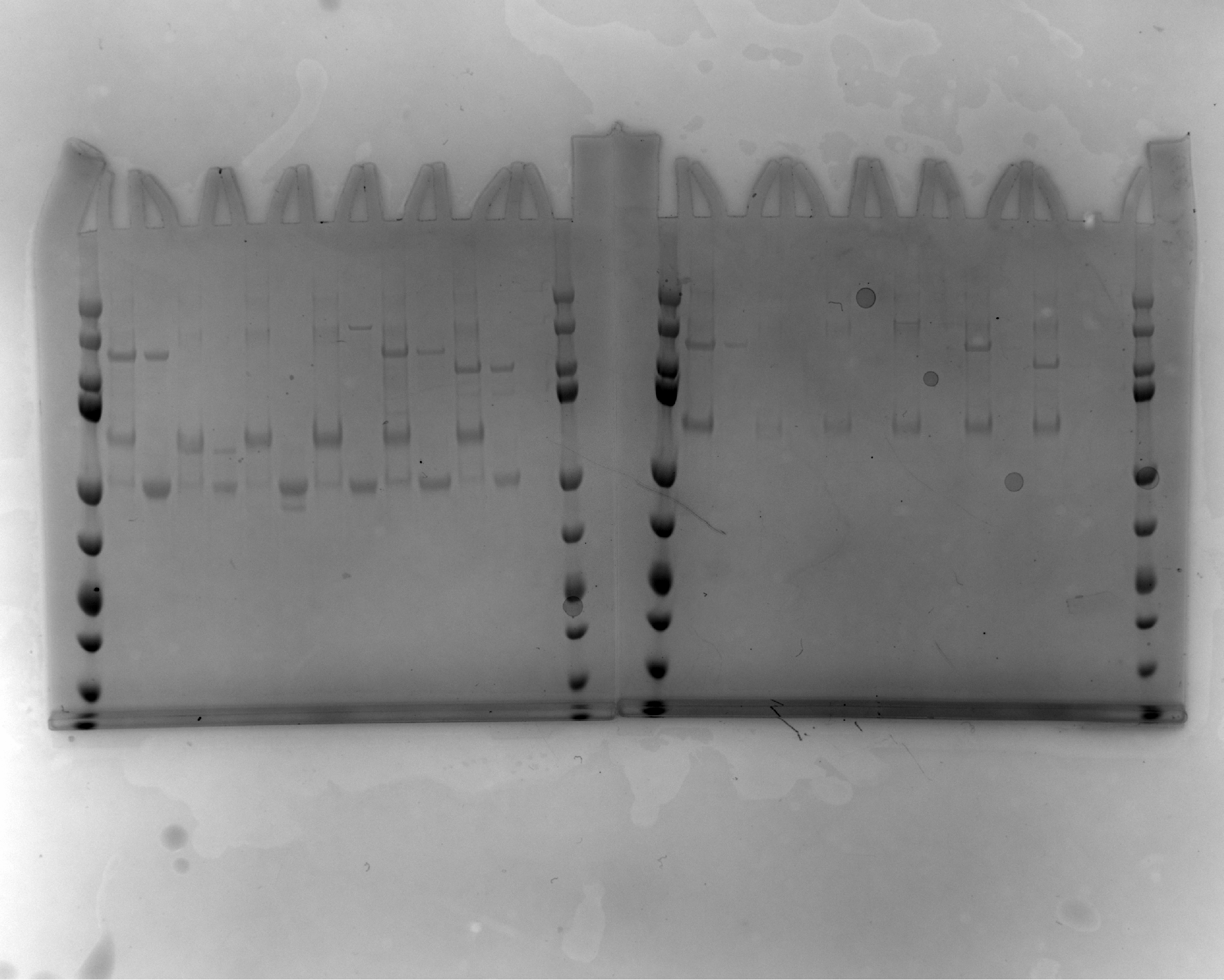

Supplement: Supplementary file 11 — Uncropped gel images. [file 41594_2024_1212_MOESM11_ESM.tif]
